# *Artemisia* spp. Essential Oils: From Their Ethnobotanical Use to Unraveling the Microbiota Modulation Potential

**DOI:** 10.3390/plants13070967

**Published:** 2024-03-27

**Authors:** Flavio Polito, Mattia Di Mercurio, Silvia Rizzo, Maura Di Vito, Maurizio Sanguinetti, Andrea Urbani, Francesca Bugli, Vincenzo De Feo

**Affiliations:** 1 Department of Pharmacy, University of Salerno, Via Giovanni Paolo II, 132, Fisciano, 84084 Salerno, Italy; fpolito@unisa.it (F.P.); defeo@unisa.it (V.D.F.); 2 Dipartimento di Scienze Biotecnologiche di Base, Cliniche Intensivologiche e Perioperatorie, Università Cattolica del Sacro Cuore, 00168 Rome, Italy; mattia.dimercurio@unicatt.it (M.D.M.); silvia.rizzo@unicatt.it (S.R.); maurizio.sanguinetti@unicatt.it (M.S.); andrea.urbani@unicatt.it (A.U.); francesca.bugli@unicatt.it (F.B.); 3 Dipartimento di Scienze di Laboratorio e Infettivologiche, Fondazione Policlinico Universitario A. Gemelli IRCCS, Largo A. Gemelli 8, 00168 Rome, Italy; 4 UOC Chimica, Biochimica e Biologia Molecolare Clinica, Dipartimento di Scienze di Laboratorio e Infettivologiche, Fondazione Policlinico Universitario A. Gemelli IRCCS, 00168 Rome, Italy

**Keywords:** *Artemisia absinthium*, *Artemisia annua*, absinthe, medicinal wine

## Abstract

Background. The 2015 Nobel Prize in Medicine, awarded for the discovery of artemisinin in *Artemisia annua*, reignited interest in aromatic plants, including *Artemisia absinthium* L. This article delves into the historical, ethnopharmacological and medicinal significance of *A. absinthium*, examining its bitter taste noted since ancient Greek times and its association with medicinal properties throughout history. Despite being banned in the 20th century due to perceived health risks; recent research has led to the reconsideration of *A. absinthium*’s potential applications. This study focuses on the prebiotic efficacy of essential oils (EOs) from two *Artemisia* species: *A. absinthium* and *A. annua*. Materials and methods. A broth microdilution test, growth curve test and *in vivo* models were used to study the impact of low doses (from 0.5% *v*/*v* to 0.00048 *v*/*v*) of *Artemisia* spp-EO on the three probiotic strains (*Lactobacillus*, *Lactobacillus casei* and *Saccharomyces boulardii*). Results. These essential oils, when used in minimal concentrations (lower than 0.06% *v*/*v*), are safe and exhibit prebiotic effects on major probiotic strains, supporting the traditional culinary use of *Artemisia* spp. Conclusion. This research opens avenues for potential applications in the food industry, emphasizing the need for further exploration into the prebiotic properties of *Artemisia* spp-EOs and their influence on the microbiota.

## 1. Introduction

The Nobel Prize in Medicine awarded in 2015 for the discovery of artemisinin, a sesquiterpenoid lactone effective in the treatment of malaria, present in *Artemisia annua* has reaccredited interest in these aromatic plants, including *Artemisia absinthium* L., which has held an important place in the history of medicine. *A. absinthium* has been known for its bitter taste since ancient Greek times where Dioscorides and Theophrastus associated it with the Greek words “ápsinthos”, i.e., unpleasant. In more recent times, Germanic literature has associated this unpleasant taste with its antiparasitic effect (“Werm” in Old German means “worm”). Over centuries, this medicinal plant has been associated with various medicinal and cosmetic properties [1]. Dioscorides identified astringent and pain-relieving properties on the gastrointestinal tract, while Plinio il Vecchio indicated its hypnotic, laxative and hair cosmetic properties, the latter demonstrating a remarkably modern perspective. In the work entitled “Physica”, the well-known Saint and Doctor of the Church named Hildegard describes [2] the effects of food on health and underlines the restorative effect of absinthe as “the most important teacher against any exhaustion”, highlighting, already in his time, the effect on the central nervous system. Even in the Renaissance, absinthe was indicated as a remedy for disorders of the gastrointestinal tract. Traditional Asian and European medicine integrated this medicinal plant not only in the treatments of gastrointestinal disorders but also in infectious diseases and insomnia. The main substances responsible for its biological activity are terpene compounds of essential oil (EO), bitter substances, flavonoids, azulenes, phenolic acids, tannins and lignans [3]. Furthermore, it is known that the antiparasitic properties of species belonging to the *Artemisia* genus, especially absinthe, were exploited by extracting the active ingredients thanks to their infusion into the wine. The earliest sources of these practices date back to Roman times, and probably as early as the time of Pythagoras. Hippocrates used this wine as a tonic and medical treatment, hence the name “Hippocratic wine”. These macerations of the *Artemisia* genus in wines have evolved over the centuries until arriving, in the 18th century, at the production of both Vermouth (in Turin by Antonio Benedetto Carpano) [4] and “Absinthe” (in Couvet by Pierre Ordinaire and Henriette Henriod), both produced with *A. absinthium* as the main aromatic plant. Historical sources document the prophylaxis against parasitic diseases through the administration of these drinks to soldiers and it was thanks to them that the use of this drink spread among the population so much so that it became the most popular drink in all social strata [5] including great artists and intellectuals [6,7]. In fact, as early as the end of the 19th century, symptoms of a pathology called “absinthe” were observed and linked to the regular consumption of this drink. Starting from the 20th century, absinthe was banned in all European nations [8]. Only in the 1990s, thanks to the Council Directive 88/388/EEC, was the use of *A. absinthium* in foods readmitted by identifying a maximum thujone limit of 35 mg/kg. Currently, drinks and foods obtained by using plants belonging to the *Artemisia* genus are still integral parts of the European culinary tradition [8].

Similarly, other species belonging to the *Artemisia* genus are also used both in traditional medicine and in nutrition. Among these, *Artemisia vulgaris* (known as river mugwort) is a Chinese plant traditionally used to treat numerous health ailments [9]. It is used not only in traditional medicine but also in cooking as a plant food to be consumed alone or as an ingredient in soups [9]. *Artemisia saharae* Pomel (a new taxon of *Artemisia herba-alba* Asso), endemic to Tunisia and Algeria, is also known as white wormwood or desert wormwood, and it is used both in medicine and in the culinary tradition [10]. *Artemisia dracunculus* (Russian dragon), closely related to French tarragon, has been consumed for centuries in food without reported adverse effects [11]. The above indicates that species belonging to the *Artemisia* genus have been used not only in traditional medicine but also in culinary practices of the European and Asian peoples. As mentioned, among the main active ingredients of *Artemisia* spp. are the terpene compounds present in EOs. Several articles have identified potential beneficial properties of these compounds for the gastrointestinal tract such as antioxidant [12,13], antimicrobial [14,15] and antiparasitic properties [16,17]. These results, on the one hand, support traditional use and, on the other hand, renew interest in the use of *Artemisia* spp. in food products. Unfortunately, even if it is known that everything ingested (eaten or drunk) influences the state of our intestinal microenvironment including the microbiota and that the latter, in turn, influences human health, very few studies have directly or indirectly evaluated the activity of extracts belonging to the *Artemisia* genus on the human intestinal microbiota. Furthermore, no study has evaluated the activity of terpene compounds of EOs on the latter. Specifically, just one study published by Li J and colleagues [18] evaluated the activity of polysaccharides from *Artemisia sphaerocephala* and two of its fractions against fecal microbiota. The authors concluded that the administration of polysaccharides from *A. sphaerocephala* could drastically modify the metabolic profile of intestinal bacteria compared to fructooligosaccharides. Mariela Martinez Davila and colleagues, in a review published in 2023, evaluated the impact of *A. absinthium* and other natural products on patients suffering from Chron’s disease. In their article, the authors highlighted that the diet supplementation of capsules containing *A. absinthium* extracts (3 × 500 mg/day) in patients receiving prednisone therapy resulted in a reduction in steroid use, starting from the second week of treatment, with consequent improvement of both clinical outcomes and quality of life [19].

This study is related to the validation of the ethnopharmacological uses of *Artemisia* spp. in traditional medicine and nutrition. The aim of the study was to evaluate the prebiotic efficacy of *Artemisia* spp. EOs to identify the impact of low doses of EO compounds potentially present in *Artemisia* spp-based foods on microbial strains belonging to the microbiota. Two types of *Artemisia* were considered: *A. absinthium*, known for its essential oils containing thujone, and *A. annua*, which lacks thujone in its EOs.

To accomplish this, we employed several methods. First, we used broth micro-dilution testing to determine the minimum inhibitory concentrations. Additionally, we conducted growth curve testing to assess how these EOs affected the growth and viability of key microbial strains like *Saccharomyces boulardii*, *Lactobacillus casei*, and *Lactobacillus rhamnosus.* This allowed us to gain insights into their prebiotic potential. Furthermore, we evaluated the safety and potential toxicity of these EOs in an *in vivo* model using *Galleria mellonella*.

By using these approaches, we sought to gain a comprehensive understanding of the prebiotic properties of *Artemisia* spp. EOs and their potential influence on the microbiota.

## 2. Results

### 2.1. Qualitative Analysis

The analysis of the EOs allowed the identification of 74 components in *A. absinthium* EO, corresponding to 96.9% of the total. The most representative class of compounds is oxygenated monoterpenes (44.6%), followed by hydrocarbon sesquiterpenes (34.8%), hydrocarbon monoterpenes (10.7%) and oxygenated sesquiterpenes (6.0%). There are also compounds that do not belong to any of the mentioned classes (0.8%). The most abundant components are trans-thujone (19.6%) and camphor (12.2%). Other components present in quantities equal to or greater than 1% include 3,6-dihydrochamazulene (7.4%), carvacrol (3.9%), geranyl-α-terpinene (3.9%), geranyl-p-cymene (3.5%), longifolene (2.8%), p-cymene (2.2%), caryophyllene oxide (2.0%), γ-terpinene (2.0%), terpinen-4-ol (1.7%), cis-sabinene hydrate (1.6%), α-terpinene (1.5%), camphene (1.4%) and cis-thujone (1.4%). In the A. annua EO, 66 components were found, corresponding to 97.8% of the total. The most representative class of compounds is that of hydrocarbon sesquiterpenes (39.6%), followed by hydrocarbon monoterpenes (26.1%), oxygenated monoterpenes (18.8%) and oxygenated sesquiterpenes (11.0%). Here, there are also compounds that do not belong to any of the mentioned classes (2.3%). The majority components are β-pinene (14.4%) and trans-chrysantenyl acetate (6.3%). Other components present in quantities equal to or greater than 1% include cis-muurola-4(14),5-diene (5.8%), α-pinene (5.4%), β-copaene (5.0%), eucalyptol (4.4%), chrysanthenol (4.3%), β-ylangene (3.9%), γ-gurjunene (3.8%), α-acorenol (3.6%), γ-eudesmol (3.2%), allo-aromadendrene (2.8%), viridiflorene (2.6%), bicyclogermacrene (2.3%), γ-terpinene (2.3%), ar-curcumene (1.7%), β-elemene (1.6%), α-farnesene (1.6%), p-cymene (1.3%), terpinen-4-ol (1.3%), γ-muurolene (1.2%), cadin-3,9-diene (1.1%), δ-elemene (1.1%), α-terpinene (1.1%), α-cadinol (1.0%), β-gurjunene (1.0%) and α-ylangene (1.0%) (Table 1).

### 2.2. Growth Curve Testing

**Figure 1:**
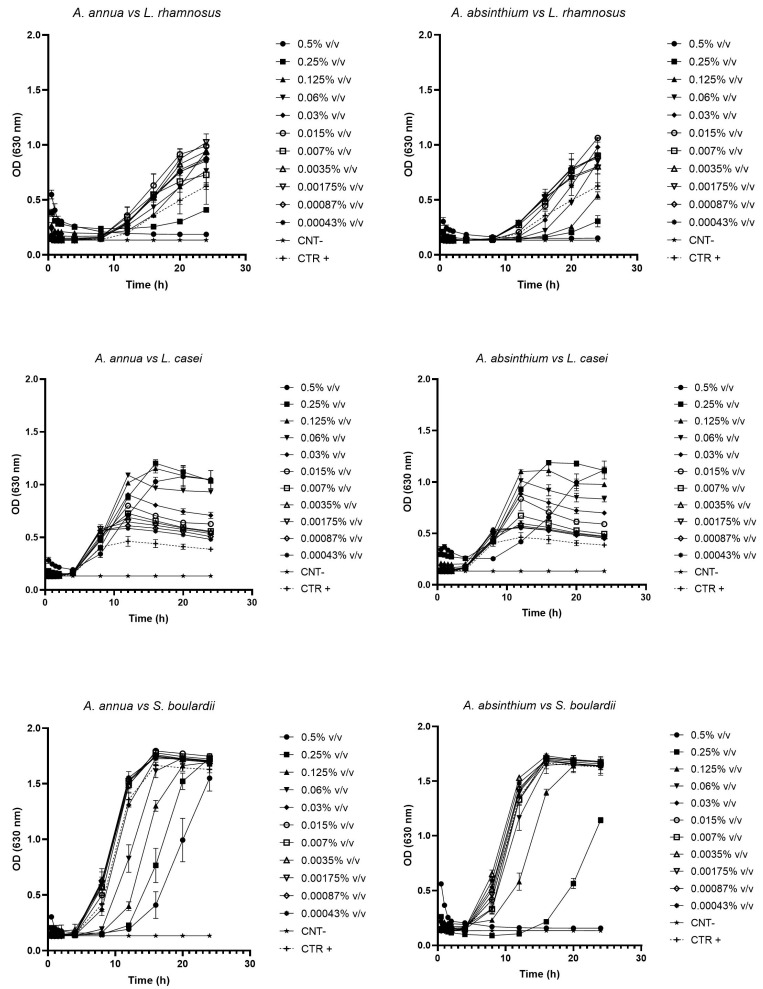
Each graph shows growth curves obtained by exposing one of the three probiotics (*L. rhamnosus*, *L. casei*, and *S. boulardii*) to the activity of AAb-EO and AA-EO. Error bars represent the standard deviation of the mean for each data point.

The test aimed at evaluating the prebiotic effectiveness of AA-EO and AAb-EO against probiotic strains showed that, starting from concentrations lower than 0.06% *v*/*v*, AA-EO was able to promote the growth of *L. rhamnosus*, while concentrations equal to or greater than 0.06% *v*/*v* had inhibitory or cytocidal activity. On the contrary, all concentrations tested against *L. casei* had growth-promoting activity. However, in the case of *S. boulardii* yeast, the analysis of growth curves did not reveal a significant probiotic effect. Concentrations lower than 0.06% *v*/*v* seemed to have no substantial impact on the growth curve, whereas values higher than or equal to 0.06% *v*/*v* exhibited growth-inhibitory activity. Similarly, AAb-EO, starting from concentrations lower than 0.06% *v*/*v*, displayed growth-promoting effects on *L. rhamnosus*. Notably, with AAb-EO, it was observed that concentrations lower than or equal to 0.0035% *v*/*v* stimulated probiotic growth, even reaching a plateau phase that was not attained within 24 h due to the slow growth of the probiotic strain in aerobic conditions. The effects of AAb-EO on both *L. casei* and *S. boulardii* mirrored those described for AA-EO (Figure 1).

**Figure 2:**
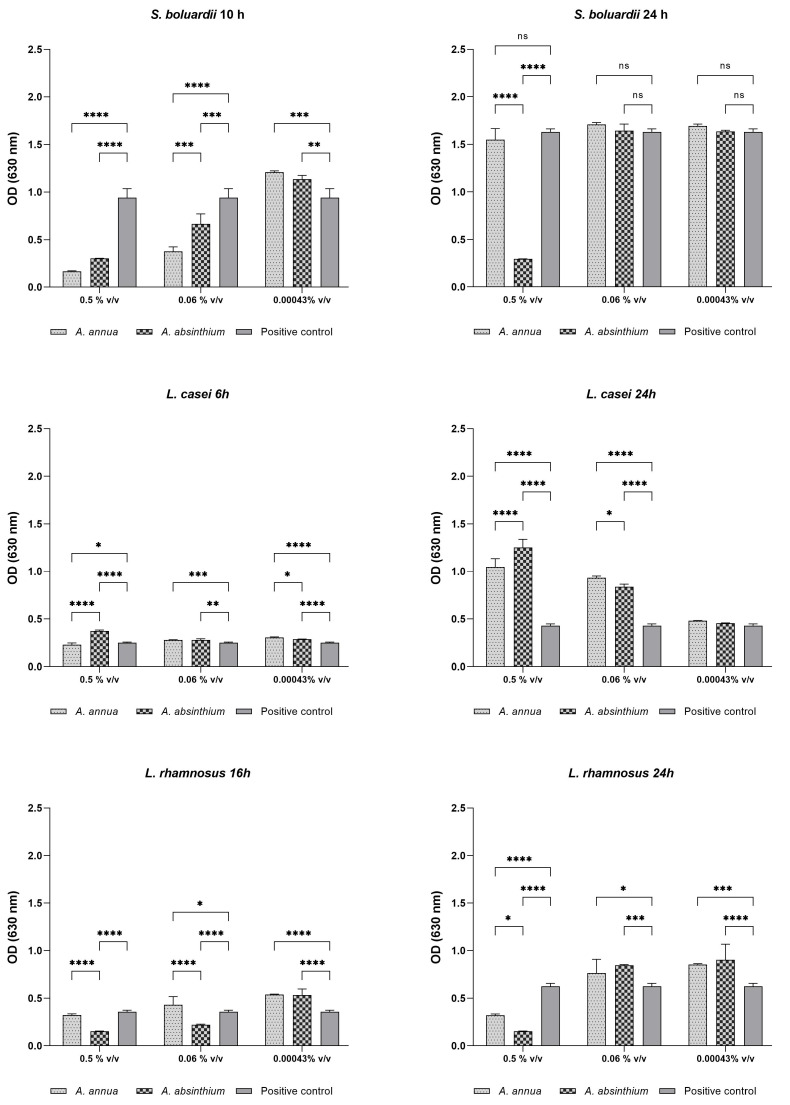
Graphs in the first column show the OD values detected at the inflection point (of the control curve) of each of the three probiotics when grown in the presence of AA-EO or AAb-EO. The graphs on the right show the OD values detected after 24 h of incubation. OD values were detected in the presence of 0.5%, 0.06%, and 0.00043% *v*/*v* of both EOs. Error bars represent the standard deviation of the mean, **** = *p* < 0.0005; *** = *p* < 0.001; ** = *p* < 0.005; * = *p* < 0.05.

The above can be well understood by observing graphs in Figure 2 that show the OD values detected both at the inflection point (different for each probiotic) and at the end-time of the incubation (24 h).

For each time point, ODs detected at the maximum concentration tested (0.5% *v*/*v*), at the middle between inhibitory and growth-promoting concentrations (0.06% *v*/*v*), and at a lowest concentration tested with prebiotic activity (0.00043% *v*/*v*) were considered. As shown in the graphs relating to *L. rhamnosus*, at the inflection point (16 h), high concentrations of AA-EO inhibited the probiotic growth more and in a statistically significant way if compared to AAb-EO (*p* < 0.0005). At the middle concentration tested (0.06% *v*/*v*), only *A. annua* statistically stimulated the growth of the probiotic compared to the control (*p* < 0.5), while at the lowest concentrations, both EOs demonstrated growth-stimulating activity. In fact, the ODs detected are statistically greater (*p* < 0.0005) than those detected in the control. This indicates that samples treated with low concentrations of EOs reached the exponential phase earlier than the control; therefore, the strain grew faster. After 24 h of incubation, in the presence of both middle and lower concentrations, OEs stimulated probiotic growth enough to reach higher OD values compared to the control. This indicates that in the plateau phase a higher CFU/mL value was detected in treated samples compared to the control. Data of *L. casei* growth indicate that at the inflection point (after 6 h of incubation), all AAb-EO concentrations showed an increase in OD values compared to the control, while the growth stimulus provided by AA-EO was significant starting from the middle concentration (*p* < 0.005). Instead, in the plateau phase, all concentrations, except the lowest tested, significantly stimulated the growth of the probiotic. Specifically, AAb-EO was more active at higher concentrations, while AA-EO was more efficient at the middle concentration. The analyses confirm that all concentrations tested accelerate the probiotic growth but only the concentrations higher than or equal to the middle ones produced a higher CFU/mL value in the plateau phase. Finally, data on *S. boulardii* growth indicate that, at the inflection point (after 10 h of growth), only low concentrations significantly stimulated (*p* < 0.005) fungal growth, while concentrations higher than 0.06% v/v strongly inhibited it. In the plateau phase, no concentration tested promoted fungal growth compared to the control; on the contrary, the maximum concentration of AAb-EO totally inhibited the strain growth.

### 2.3. Cell Viability

**Figure 3:**
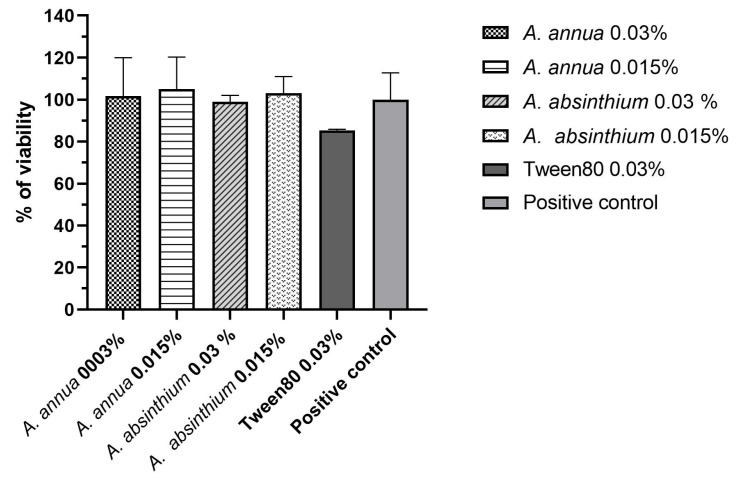
In vitro toxicity. The figure shows the viability (%) of Caco-2 cells after the treatment of AAb-EO (0.03% *v*/*v*) and AA-EO (0.015% *v*/*v*). Error bars represent the standard deviation of the mean.

Figure 3 presents the evaluation of Caco-2 cell viability using the AlamarBlue assay, specifically focusing on the two higher concentrations of AA-EO and AAb-EO, which did not exhibit any inhibitory activity. Our results demonstrate that none of the concentrations within the range of 0.03% to 0.0007% *v*/*v* led to a reduction in cell viability during the entire incubation period. Additionally, Tween 80, even at its maximum concentration tested, did not display any toxicity. Furthermore, it is noteworthy that no treatments showed any statistically significant differences when compared to the untreated positive control (*p* > 0.5). This indicates that these concentrations and treatments did not adversely affect Caco-2 cell viability, emphasizing their safety and non-toxic nature.

### 2.4. In Vivo Toxicity

**Figure 4:**
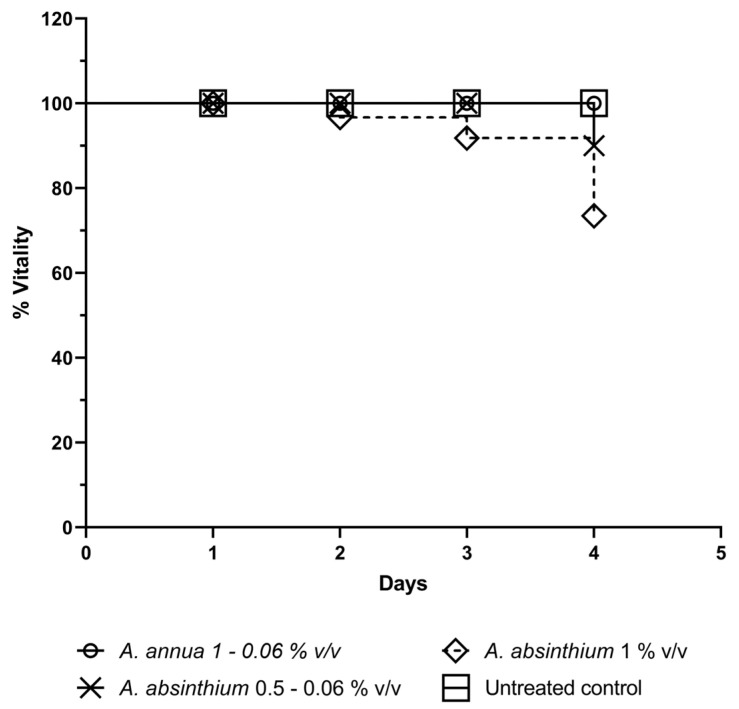
*In vivo* toxicity. The figure shows the viability (%) of *Galleria mellonella* larvae treated with a scalar dilution of AAb-EO, AA-EO or untreated (rhombus).

Data obtained from cytotoxicity tests (Figure 4) showed no mortality in all groups treated with scalar concentrations of AA between 1 and 0.06% *v*/*v*. Treatment with similar concentrations of AAb equal or lower than 0.5% *v*/*v* showed a 10% of toxicity only 4 days after treatment, while the group treated with a concentration equal to 1% *v*/*v* showed 10% toxicity after 3 days, which grew to 20% on the fourth day. The observed differences in toxicity with the tested concentrations of AAb were not statistically different.

## 3. Discussion

The chemical composition reported in this work differs in part from what is reported in the literature, especially regarding the main compounds. The comparison with works concerning EOs from Tunisian AAbs [20,21,22] highlighted the presence of the same main components (*trans*-thujone, camphor and chamazulene) but in different quantities. The *trans*-thujone in the present work has a higher concentration than the EOs analyzed in the literature, while camphor and chamazulene have lower concentrations. Finally, a recent review [23] highlighted that the characteristic compounds of AAb-EO are bornyl acetate, cadinene, chamazulene, camphene, camphor, linalool, myrcene, *trans*-sabinyl acetate, γ-terpinene, 4-terpineol, *cis-* and *trans-*thujone. Of these, many are present in the EOs studied in this work, and some of these were the main components: *trans-*thujone, camphor, chamazulene, γ-terpinene, terpinen-4-ol, camphene and *cis*-thujone (1.4%). The genetic background, environmental conditions [24] and harvesting of the plant far from the flowering period can explain the different concentrations [25]. In the literature, there are several contributions regarding the composition of AA-EOs from Italy. All these works highlight great variability both in the type of compounds and in their concentrations [26,27]. A 2014 review highlighted the main components present in AA-EO [28] across various regions of the planet: artemisia alcohol, artemisia ketone, borneol, camphene, camphene hydrate, camphor, *trans*-caryophyllene, chrysanthenone, eucalyptol, β-farnesene, germacrene D, α-guaiene, linalool, myrcene, α-pinene, *trans*-pinocaerveol, sabinene, and spathulenol. Among these, only some compounds were present in the EOs studied in this work, but with different percentages: α-pinene, eucalyptol, spathulenol, camphor, germacrene D, camphene, and linalool. Despite the great variety in compositions, all studies have in common the absence, or a very low concentration, of components recognized as neurotoxic such as thujone and camphor [29,30,31] This allows for safer use of AA-EO in formulations intended for human use such as the consumption of processed foods or prebiotic-based functional foods.

The culinary use of species belonging to the *Artemisia* genus has been known for centuries in European populations. This was possible because these plants are edible species that grow spontaneously in these geographic areas. In a recent review, Pereira and collaborators [32] argued that this type of plant is acquiring more and more importance for the food industry both because consumers increasingly demand a product of natural origin and also because they are cheaper to produce than cultivated plants. In fact, they are better suited to adapt to different climatic conditions and biotic and abiotic factors. Furthermore, it is known that aromatic plants have “accompanied” the production of traditional dishes over the centuries, not only giving them their characteristic flavor but also small quantities of bioactive molecules with beneficial properties for the body’s defense and food preservation. One such example is the use of *Cinnamomum zeylanicum* Blume from bark (cinnamon) to produce sweet creams widely used in the south of Italy. The introduction of small quantities of cinnamon gives a pleasant aroma to the pastry cream and preserves it from the rapid deterioration that is inevitable at the higher southern temperatures. Another example is mulled wine, already known to Roman and Greek times with its ancestor named “conditum paradoxum” and produced to this day in some cold European areas including northern Italy. This type of hot medicinal wine, still traditionally used today, was joined in the Middle Ages by cold medicinal wines. The latter are known as enoliths (a pharmaceutical formula that involves the maceration of medicinal plants in wine) and, although already known in Galeno times, these had their maximum diffusion with monasticism in the Middle Ages in which they became an integral part of monastic medicine.

Eleven medicinal plants were used in the production of Hippocras, a spicy wine that found its place in European courts over the centuries. Sources relating to the production of this alcoholic drink are found in various medieval sources with small variations in which the same medicinal plants were always used. A new version of hippocras without the addition of honey appears with the noblewoman Isabella De Medici Orsini whose recipe has come down to us thanks to the painstaking historical recovery intervention of Dr. Sandra Ianni and the formulation of Marco Sarandrea, expert herbalist and manager of a small Italian herbal company named Sarandrea [33]. As discussed extensively in the introduction, Vermouth and Absinthe are also enoliths, created, respectively, by Andonio Benedetto Carpano and Pierre Ordinaire and Henriette Henriod. These fortified wines, obtained during the war period through the maceration of medicinal plants like those of the *Artemisia* genus in wines, served as substitute for expensive French wines and relieved the suffering of soldiers at war. Since the 19th century, both medicated wines and the use of *Artemisia* spp. underwent an inflection. The use of *Artemisia* spp. in alcoholic beverages had an inflection throughout the 20th century until the EC reinstated their controlled use in foods after excluding that the neurotoxic activity of alcoholic beverage was attributable to it. In contrast, the current trend shows a continual increase in the use of medicinal plants in food and drink production due to the need to produce increasingly high-performance and beneficial foods based to recent scientific evidence.

Although the antimicrobial, antiparasitic and antioxidant properties of terpenic compounds obtained from *Artemisia* spp. EOs [14], including AA-EO and AAb-EO, are known, nothing is known regarding their impact on the intestinal microbiota when used in the culinary tradition, as food additives or used in the formulation of functional foods. In a recent review, Zhou X and colleagues [34] highlighted that the long-term exposure to natural or synthetic additives could induce changes in the microbiota responsible for various pathologies; on the other hand, there is a large body of literature indicating that the careful choice of natural additive could confer beneficial effects for the intestinal microbiota. Therefore, the study of the impact of natural additives obtained from aromatic plants on the microbiota or on probiotic strains used in traditional or functional foods is important both to validate their traditional use in cooking and to select the best spices to include as extracts in food and drinks.

This study focuses on assessing the prebiotic activity of AA-EO and AAb-EO derived from two *Artemisia* species in order to identify the impact of low doses of the terpene compounds in EOs, such as those potentially present in *Artemisia* spp-based foods, on microbial strains belonging to the microbiota. In fact, the use of the vegetal matrix as an ingredient in food products, or the processing of this through various extraction methods (e.g., alcoholic or hydroalcoholic maceration) used to produce drinks, involves the presence of small quantities of essential oils (or their terpene compounds) in food products for which the activity on microbial strains belonging to the microbiota is not known. The efficacy of AA-EO and AAb-EO was tested against three probiotic strains (*L. casei*, *L. rhamnosus*, and *S. boulardii*) commonly employed in the production of processed foods, food supplements, and the development of functional foods. In their review, Bottari et al. elucidate the prevalence of *Lactobacillus* spp., specifically *L. casei*, *Lactobacillus paracasei*, and *L. rhamnosus*, as prominent species in dairy products [35]. The extended ripening duration further contributes to their dominance, shaping the distinctive characteristics of the food products [35]. Furthermore, some species of the *Saccharomyces* genus, such as *S. cerevisiae* var *boulardii*, are used together with other probiotic species in the production of non-dairy foods. Through the fermentation processes, these probiotics release and/or modify secondary metabolites capable of increasing the quality of foods and drinks [36].

The results of this article show that the lowest concentrations of both EOs (0.00043% *v*/*v*) are able to statistically accelerate (*p* < 0.0005) the growth rate of all three probiotic strains. However, low concentrations do not necessarily lead to greater probiotic growth. Indeed, at the minimum tested concentration, higher optical density (OD) values were observed at the conclusion of the 24 h incubation period. Specifically, this increase in OD values was only discernible in the case of *L. rhamnosus*, corresponding to a higher colony-forming unit per milliliter (CFU/mL) count compared to the control. In all other cases, in the presence of the stimulus of the EOs, the probiotic strains reach the same OD values as the control, although in less time. Instead, results show that to have both effects on *L. casei*, it is necessary to use higher concentrations of EOs (0.06% *v*/*v*), while none of the concentrations tested against *S. boulardii* produce both effects. Finally, it is interesting to note that the greatest variation in CFU/mL is given using EOs against *L. casei* and that this stimulus is directly proportional to the EO concentration. Therefore, it is possible to conclude that even if each probiotic strain responds differently to low concentrations of EOs, very low concentrations of these EOs (0.00043% *v*/*v*) do not harm the probiotic activity but rather promote it. The European Medicine Agency monograph [37] reported that the concentration of EO in the fresh *A. absinthium* plant varies between 0.2% and 1.5%. Considering that this plant is used in small quantities in cooking to flavor foods and drinks, the final concentration of EO terpene compounds present in these *Artemisia*-based products would be very low. Therefore, it is possible to conclude that the traditional use of *A. absinthium* in culinary practices is not capable of damaging probiotic strains, but it can potentially promote probiotics growth without having toxic effects (Figure 3 and Figure 4). What has been said is even more reasonable if we consider the use of *A. annua* that, as shown by the chemical analysis, has minimal or no concentrations of neurotoxic components. This observation, coupled with the traditional use of such aromatic plants, not only reinforces their credibility but also paves the way for compelling possibilities in the field of nutrition. It presents opportunities for the development of food supplements or functional foods incorporating probiotics and *Artemisia*. This redemption of the *Artemisia* genus, historically a subject of diverse scientific debates, holds promise for various applications in the food industry.

## 4. Materials and Methods

### 4.1. Plant Material

Samples of leafy young stems of *A. absinthium* (AAb) and *Artemisia annua* (AA) were collected, respectively, in the territory of the city of Beja (Tunisia) during October 2022 and in a field located in the municipality of Bracigliano, in the province of Salerno in Campania, during June 2023. Both aromatic plants were identified by Prof. V. De Feo.

### 4.2. Extraction of Essential Oils

According to the European Pharmacopoeia [38], fresh branches and leaves were reduced to fragments and then subjected to hydro-distillation for 3 h using an Albrigi-Inherba “Extractor plus 20 L” hydrodistiller model. The EOs were dissolved in n-hexane, dried over anhydrous sodium sulfate, and kept under N_2_ at 4 °C in the dark until analysis. Distillation of AAb produced 2.53 × 10^3^ g of distillate containing 1.77 g of EO (yield = 0.07%). The distillation of AA produced 0.5 × 10^3^ g of distillate containing 0.57 g of EO (yield = 0.11%)

### 4.3. Analysis of Essential Oils

Analytical gas chromatography was conducted on a Perkin–Elmer Sigma-115 gas chromatograph accessorized with an FID and a data handling processor. The separation was obtained with an HP-5MS fused-silica capillary column (30 m × 0.25 mm i.d., 0.25 μm film thickness). Column temperature: 40 °C, with 5 min initial hold, then increasing to 270 °C at 2 °C/min, 270 °C (20 min); splitless injection (1 μL of a 1:1000 n-hexane solution). The injector and detector temperatures were 250 and 290 °C, respectively. Analysis was also run by using a fused silica HP Innowax polyethylenglycol capillary column (50 m × 0.20 mm i.d., 0.25 μm film thickness). In both cases, He was employed as a carrier gas (1.0 mL/min). GC–MS analyses were conducted with a Hewlett–Packard 5890 A gas chromatograph linked online to an HP mass selective detector (MSD 5970HP) equipped with a DB-5 fused-silica column (25 m × 0.25 mm i.d.; 0.33 μm film thickness). The ionization energy voltage was 70 eV and the electron multiplier energy was 2000 V. The gas-chromatographic conditions were as described above; transfer line 295 °C. Most of the components were identified by comparing their Kovats indices (Ki) with those of the literature [39,40,41,42]. Furthermore, a careful analysis of the mass spectra compared to those of pure compounds available in our laboratory or to those present in the NIST 02 and Wiley 257 mass libraries [43] was found. The Kovats indices were determined in relation to a homologous series of n-alkanes (C10-C35), under the same operating conditions. For some compounds, the identification was confirmed by co-injection with standard samples. Components’ relative concentrations were calculated using peak area normalization. Response factors were not considered.

### 4.4. Bacterial Strains, Culture Medium and Chemicals

The prebiotic effectiveness of EOs was tested against three probiotics reference strains: *S. boulardii* (CBS5926), *L. casei* (R0215) and *L. rhamnosus* (DSM20021). MRS (VWR International Srl, Milan, Italy) and Sabouraud (Sigma-Aldrich, Saint Louis, MO, USA) mediums were used for microbiological testing carried out with bacterial and fungal strains, respectively. Tween 80 (Fisher Scientific, Geel, Belgium) surfactant was used as an emulsifier in microbiological tests.

### 4.5. Prebiotic Activity of EOs

For the assessment of the prebiotic activity of EOs, an EO solution was prepared by blending EOs with Tween 80 (Fisher Scientific, Geel, Belgium) surfactant in a *v*/*v* ratio equal to 1:1. The mixture was then adjusted to the final volume using LB medium broth for *Lactobacillus* and Sabouraud medium broth (Sigma-Aldrich, Saint Louis, MO, USA) for yeast. To initiate the experiment, 50 µL of the EO solution was dispensed into each well of a 96-well plate. Serial dilutions were subsequently performed to obtain a final concentration ranging from 0.5% *v*/*v* (5 μL/mL) to 0.00048% *v*/*v* (0.0048 µL/mL). A 0.5 McFarland suspension was adjusted to achieve a microbial density of 1 × 10^6^ colony-forming units (CFU)/mL, and 50 µL of this suspension was inoculated into each respective well. A positive control (without EO), sterility control (with only growth medium) and negative control (with growth medium and Tween 80) were included. The plates were then incubated at 37 °C for 24 h. After the incubation period, the impact of the essential oil treatment on the probiotic strains was assessed by analyzing the growth curves kinetics using a Cytation 5 instrument. All tests were performed in triplicate.

### 4.6. Growth Curve Testing

To study the prebiotic efficacy of the *Artemisia* spp-EOs, *L. rhamnosus*, *L. casei* and *S. boulardii* were tested at scalar concentrations with EOs, between 0.5% *v*/*v* (5 μL/mL) and 0.00048 *v*/*v* (0.0048 µL/mL) in LB culture medium broth (Oxoid, Basingstoke, Hampshire, UK) for lactobacilli and Sabouraud for yeast. The growth curve involves a spectrophotometric investigation in which the increasing optical density (OD) of microorganisms in culture media is compared to their proliferation. To evaluate the prebiotic effect of *Artemisia* OEs, a flat-bottom 96-well plate was prepared as mentioned in Section 4.5. Plates were incubated in a Cytation 5 Cell Imaging Multi-Mode Reader (Agilent, Santa Clara, CA, USA). The incubation was performed at 37 °C and 5% CO_2_ on an orbital continuous shaker at 205 rpm. The optical density was measured at ʎ = 630 nm every hour for 24 h. All assays were performed in duplicate with a positive and negative control.

### 4.7. Cell Culture

Caco-2 cells from passage 5 to 20 were cultured in 25 cm^2^ surface area flasks (Corning Inc. Life Sciences, New York, NY, USA) using Dulbecco’s modified Eagle’s medium (DMEM, Gibco, ThermoFisher Scientific, Waltham, MA, USA) supplemented with 20% fetal bovine serum (FBS, Gibco, ThermoFisher Scientific, Waltham, MA, USA), 1% L-glutammine 200 mM (Gibco, ThermoFisher Scientific, Waltham, MA, USA), 1% penicillin–streptomycin 10,000 U/mL (Gibco, ThermoFisher Scientific, Waltham, MA, USA) and maintained at 37 °C under 5% CO_2_. Cells were seeded at 104 cells/cm^2^ in 96-well plates (Corning Inc. Life Sciences, New York, NY, USA) for cytotoxicity assay.

### 4.8. Cell Viability

Cell viability was assessed using the alamarBlue assay (ThermoFisher Scientific, Waltham, MA, USA). Human epithelial cells were cultured in DMEM medium and treated with decreasing concentrations of *Artemisia annua* and *Artemisia absinthium* EOs (0.03% *v*/*v* to 0.0007% *v*/*v*) for 24 h. The EOs were dissolved in Tween 80 with a ratio of 1:1 and then diluted in culture medium. After 24 h, cells were washed with PBS and incubated with 10% (*v*/*v*) of alamarBlue reagent for 3 h at 37 °C. Fluorescence was measured at 560 nm excitation and 590 nm emission using a Cytation 5 Cell Imaging Multi-Mode Reader (Agilent, USA). Untreated cells were used as positive controls for 100% viability. To evaluate the toxicity of Tween 80, experiments containing only culture medium and Tween 80 at the highest concentration tested (0.03% *v*/*v*) were performed. Three different experiments were performed in triplicate, and the cell viability was calculated as follows:

% viability = (Treated cells RFU)/(Untreated cells RFU) × 100

### 4.9. In Vivo Toxicity Study

The toxicity of *Artemisia* spp-OEs was assessed by using an *in vivo Galleria mellonella* model. *Larvae* were maintained at 30 °C in an aerobic incubator, and those displaying any color changes in their bodies were excluded from the study. Larvae were treated with scalar concentrations of EOs ranging from 1000 to 63 µg/mL (corresponding to 1% and 0.06% *v*/*v*). This was achieved by injecting 10 μL of the oil solution (in physiological solution) into the haemocoel through the last left pro-leg of ten larvae, using a 0.5 mL syringe. Prior to administration, the injection site was decontaminated with 70% ethanol. The larvae were then monitored at 24 h intervals over a span of 96 h to evaluate any effects or changes.

## 5. Conclusions

In conclusion, data from this work indicate that terpene compounds present in both phytocomplex of *Artemisia* spp-EOs, if used in very low concentrations (from 0.5% *v*/*v* to 0.00048 *v*/*v*) such as those present in foods or drinks produced with plants belonging to the *Artemisia* genus, are safe for use and exert a prebiotic effect on the main probiotic strains present in foods or functional foods. If further confirmed, these data support the traditional culinary use of *Artemisia* spp. and open a very interesting scenario for their use in the food industry.

## Figures and Tables

**Table 1:** Chemical volatile composition of *A. absintium* and *A. annua* EOs.

N	Compound Name	AAb-EO	AA-EO	KI ^a^	KI ^b^	Identification ^c^
		*%*	*%*			
1	1-Ethyl-2-methylcyclopentane	-	0.1	761	-	1,2
2	*trans*-7-Methyl-3-octene	-	0.1	797	-	1,2
3	1-Nonene	*-*	0.4	823	-	1,2
4	Tricyclene	0.1	-	843	1047	1,2
5	α-Thujene	0.1	0.2	850	-	1,2,3
6	α-Pinene	0.8	5.4	854	1036	1,2,3
7	Camphene	1.4	0.2	866	1075	1,2,3
8	β-Pinene	0.6	14.4	889	1120	1,2,3
9	Myrcene	0.2	0.2	909	1145	1,2
10	α-Phellandrene	0.4	0.1	917	1177	1,2,3
11	α-Terpinene	1.5	1.1	929	1170	1,2,3
12	*p*-Cymene	2.2	1.3	937	1250	1,2
13	Limonene	0.3	-	940	1180	1,2,3
14	Eucalyptol	0.6	4.4	941	1210	1,2,3
15	*cis*-Ocimene	0.2	0.1	954	1225	1,2
16	γ-Terpinene	2.0	2.3	970	1221	1,2,3
17	*cis*-Sabinene hydrate	1.6	-	977	-	1,2
18	Terpinolene	0.7	0.5	996	1289	1,2,3
19	α-Pinene oxide	0.1	-	1003	1384	1,2
20	*cis*-Thujone	1.4	-	1008	1430	1,2
21	Linalool	-	0.2	1009	1506	1,2,3
22	1,3,8-*p*-Menthatriene	0.2	-	1013	-	1,2
23	*trans*-Thujone	19.6	-	1021	1442	1,2
24	*cis*-p-Menth-2-en-1-ol	0.4	-	1028	-	1,2
25	*trans*-Chrysanthenol	-	0.1	1030	-	1,2
26	*allo*-Ocimene	-	0.3	1035	1382	1,2
27	Camphor	12.2	0.6	1049	1491	1,2,3
28	Isopulegol	0.1	-	1050	1533	1,2
29	*cis*-Chrysanthenol	0.3	4.3	1052	-	1,2
30	Eucarvone		0.1	1061	-	1,2
31	Pinocarvone	0.1	-	1062	-	1,2
32	Borneol	0.7	-	1067	1715	1,2
33	*neoiso*-Isopulegol	0.1	-	1073	-	1,2
34	Terpinen-4-ol	1.7	1.3	1079	1636	1,2
35	*trans*-Isocitral	0.1	-	1084	-	1,2
36	α-Terpineol	0.3	0.8	1092	1662	1,2,3
37	*cis*-Piperitol	0.1	-	1095	-	1,2
38	*trans*-4-Caranone	0.3	-	1100	-	1,2
39	*trans*-Piperitol	0.2	-	1102	1690	1,2
40	2,6-Dimethyl-2-vinyl-5-heptenoic acid	0.1	-	1118	-	1,2
41	1-(3-methylbutyl)-Cyclopentene	0.1	-	1128	-	1,2
42	Cumin aldehyde	0.1	-	1133	1802	1,2
43	Carvotanacetone	0.5	-	1138	1697	1,2
44	*trans*-Chrysanthenyl acetate	-	6.3	1156	-	1,2
45	Perilla aldehyde	0.1	-	1165	1785	1,2
46	Bornyl acetate	-	0.2	1176	1575	1,2
47	Carvacrol	3.9	0.4	1195	2219	1,2,3
48	δ-Elemene	0.2	1.1	1218	1479	1,2
49	*trans*-Carvyl acetate	-	0.1	1225	-	1,2
50	*α-*Longipinene	-	0.5	1228	-	1,2
51	α-Cubebene	0.1	-	1230	1445	1,2
52	Ionene	0.1	-	1233	-	1,2
53	Silphiperfol-5,7(14)-diene	-	0.1	1236	-	1,2
54	α-Ylangene	-	1.0	1251	1492	1,2
55	α-Copaene	0.6	0.3	1254	1477	1,2
56	β-Bourbonene	0.6	0.2	1262	1498	1,2
57	β-Cubebene	0.1	-	1269	1525	1,2
58	β-Elemene	0.4	1.6	1272	-	1,2
59	γ,4-dimethyl-Benzenebutanal	0.2	-	1284	-	1,2
60	Longifolene	2.8	-	1295	1574	1,2
61	α-Cedrene	0.1	-	1297	-	1,2
62	β-Cedrene	0.4	0.2	1298	1587	1,2
63	β-Ylangene	-	3.9	1299	1492	1,2
64	β-Copaene	0.1	5.0	1313	-	1,2
65	β-Gurjunene	-	1.0	1320	1655	1,2
66	Aromandendrene	0.4	0.3	1321	1631	1,2
67	*cis*-Cadina-1(6),4-diene	0.1	-	1332	-	1,2
68	Propanoic acid, 2-methyl-, 1,7,7-trimethylbicyclo[2.2.1]hept-2-yl ester, exo-	0.2	-	1337	-	1,2
69	α-Himachalene	-	0.4	1340	-	1,2
70	β-Chamigrene	0.9	-	1345	1724	1,2
71	*allo*-Aromadendrene	-	2.8	1347	1660	1,2
72	Germacrene D	0.8	0.3	1349	1712	1,2
73	cis-Muurola-4(14),5-diene	-	5.8	1352	-	1,2
74	β-Selinene	0.6	-	1353	1725	1,2
75	γ-Gurjunene	-	3.8	1355	-	1,2
76	β-Ionone	0.1	-	1358	1907	1,2
77	γ-Muurolene	-	1.2	1366	1725	1,2
78	*ar*-Curcumene	-	1.7	1370	1786	1,2
79	*α-*Farnesene	-	1.6	1383	1752	1,2
80	Cadina-3,9-diene	-	1.1	1393	-	1,2
81	Elemol	0.5	-	1413	2076	1,2
82	Viridiflorene	-	2.6	1415	-	1,2
83	α-Cedrene epoxide	0.1	-	1417	-	1,2
84	Bicyclogermacrene	-	2.3	1432	1756	1,2
85	Spathulenol	0.4	0.8	1439	-	1,2
86	Caryophyllene oxide	2.0	-	1442	2008	1,2
87	*trans*-β-Guaiene	-	0.6	1451	-	1,2
88	Salvial-4(14)-en-1-one	0.5	-	1452	2037	1,2
89	Aristolene epoxide	-	-	1453	-	1,2
90	*trans*-γ-Bisabolene	-	0.2	1465	-	1,2
91	Humulene epoxide II	0.2	-	1466	-	1,2
92	Junenol	0.6	-	1474	-	1,2
93	Guaiol	-	0.7	1488	2094	1,2
94	3,6-Dihydrochamazulene	7.4	-	1489	-	1,2
95	γ-Eudesmol	-	3.2	1492	2178	1,2
96	Ylangenol	0.3	-	1494	-	1,2
97	α-Acorenol	-	3.6	1498	-	1,2
98	1-*epi*-Cubenol	0.1	-	1500	-	1,2
99	β-Eudesmol	0.7	0.7	1500	2215	1,2
100	α-Cadinol	-	1.0	1522	2224	1,2
101	Cedr-8(15)-en-10-ol	0.3	-	1523	-	1,2
102	Aromadendrene oxide	-	0.5	1535	-	1,2
103	Germacra-4(15),5,10(14)-trien-1-α-ol	0.3	-	1537	-	1,2
104	α-Bisabolol	-	0.5	1538	2232	1,2
105	Chamazulene	11.7	-	1581	-	1,2,3
106	6,10,14-trimethyl-2-Pentadecanone	0.2	-	1691	2131	1,2
107	Geranyl-α-terpinene	3.9	-	1834	-	1,2
108	Geranyl-*p*-cymene	3.5	-	1837	-	1,2
109	Campesterol acetate	-	0.1	2697	-	1,2
110	Olean-12-en-3β-ol acetate	-	0.1	2685	-	1,2
111	3β-Stigmasta-5,22-dien-3-ol acetate	-	0.2	2709	-	1,2
112	Stigmastan-3,5,22-trien	-	0.2	2745	-	1,2
113	β-Sitosterol acetate	-	0.9	2766	-	1,2
114	5β-Cholestan-3-one, cyclic ethylene acetal	-	0.2	2987	-	1,2
	Total	96.9	97.8			
	Monoterpenes hydrocarbons	10.7	26.1			
	Oxygenated monoterpenes	44.6	18.8			
	Sesquiterpenes hydrocarbons	34.8	39.6			
	Oxygenated sesquiterpenes	6.0	11.0			
	Others	0.8	2.3			

^a^ Kovats index determined relative to the Rt of a series of n-alkanes (C10–C35) on an HP-5MS column; ^b^ Kovats index on the polar HP Innowax capillary column; ^c^ Identification method: 1 = linear retention index; 2 = identification based on the comparison of mass spectra; 3 = Co-injection with standard compounds. All pure compounds were purchased from Sigma-Aldrich, St. Louis, MO, USA (Merck Spa): α-thujene (≥95.0%), α-pinene (98%), camphene (95%), β-pinene (99%), α-phellandrene (≥95.0%), α-terpinene (≥95.0%), limonene (97%), eucalyptol (99%), γ-terpinene (97%), terpinolene (≥95%), linalool (97%), camphor (96%), α-terpineol (≥96%), carvacrol (98%), chamazulene (≥95.0%); - = absent. AAb-EO = *A. absinthium* EO, AA-EO = A. annua EO.

## Data Availability

Data are contained within the article.
